# Germline genes hypomethylation and expression define a molecular signature in peripheral blood of ICF patients: implications for diagnosis and etiology

**DOI:** 10.1186/1750-1172-9-56

**Published:** 2014-04-17

**Authors:** Guillaume Velasco, Emma L Walton, Delphine Sterlin, Sabrine Hédouin, Hirohisa Nitta, Yuya Ito, Fanny Fouyssac, André Mégarbané, Hiroyuki Sasaki, Capucine Picard, Claire Francastel

**Affiliations:** 1 Université Paris Diderot-Paris7, CNRS UMR7216, Epigénétique et Destin Cellulaire, Case Courrier 7042; 35, rue Hélène Brion, 75205 Paris, France; 2 Center for the Study of Primary Immunodeficiencies, Assistance Publique-Hôpitaux de Paris, Necker Hospital, Paris, France; 3 Institut National de la Santé et de la Recherche Médicale, Laboratory of Human Genetics of Infectious Diseases, Necker Branch, U980 Paris, France; 4 Imagine Institute, University Paris Descartes, Sorbonne Paris Cité, Paris, France; 5 Division of Epigenomics and Development, Department of Molecular Genetics, Medical Institute of Bioregulation, Kyushu University, Fukuoka, Japan; 6 Service d'Hémato-oncologie Pédiatrique et Transplantations Médullaires, Centre Hospitalier Universitaire de Nancy, Hôpital Brabois Enfants, Vandœuvre-lès-Nancy, France; 7 Unité de Génétique Médicale et laboratoire associé, INSERM UMR S_910, Faculté de Médecine, Université Saint Joseph, Beirut, Lebanon

**Keywords:** Agammaglobulinemia, DNA methylation, Heterochromatin, DNA, Satellite, Gene expression, Biological markers/diagnosis use, Biological markers/etiology, Genes, X-linked

## Abstract

**Background:**

Immunodeficiency Centromeric Instability and Facial anomalies (ICF) is a rare autosomal recessive disease characterized by reduction in serum immunoglobulins with severe recurrent infections, facial dysmorphism, and more variable symptoms including mental retardation. ICF is directly related to a genomic methylation defect that mainly affects juxtacentromeric heterochromatin regions of certain chromosomes, leading to chromosomal rearrangements that constitute a hallmark of this syndrome upon cytogenetic testing. Mutations in the *de novo* DNA methyltransferase DNMT3B, the protein ZBTB24 of unknown function, or loci that remain to be identified, lie at its origin. Despite unifying features, common or distinguishing molecular signatures are still missing for this disease.

**Method:**

We used the molecular signature that we identified in a mouse model for ICF1 to establish transcriptional biomarkers to facilitate diagnosis and understanding of etiology of the disease. We assayed the expression and methylation status of a set of genes whose expression is normally restricted to germ cells, directly in whole blood samples and epithelial cells of ICF patients.

**Results:**

We report that DNA hypomethylation and expression of *MAEL* and *SYCE1* represent robust biomarkers, easily testable directly from uncultured cells to diagnose the most prevalent sub-type of the syndrome. In addition, we identified the first unifying molecular signatures for ICF patients. Of importance, we validated the use of our biomarkers to diagnose a baby born to a family with a sick child. Finally, our analysis revealed unsuspected complex molecular signatures in two ICF patients suggestive of a novel genetic etiology for the disease.

**Conclusions:**

Early diagnosis of ICF syndrome is crucial since early immunoglobulin supplementation can improve the course of disease. However, ICF is probably underdiagnosed, especially in patients that present with incomplete phenotype or born to families with no affected relatives. The specific and robust biomarkers identified in this study could be introduced into routine clinical immunology or neurology departments to facilitate testing of patients with suspected ICF syndrome. In addition, as exemplified by two patients with a combination of molecular defects never described before, our data support the search for new types of mutations at the origin of ICF syndrome.

## Background

Immunodeficiency with centromeric instability and facial anomalies (ICF; OMIM no. 242860) is a rare autosomal recessive disorder mainly characterized by primary immunodeficiency [1]. Recurrent infections are the presenting symptom, usually in early childhood. Other features include mild facial anomalies and variable symptoms including intellectual disability, congenital malformations and developmental delay [2].

ICF syndrome is a genetically heterogeneous disease with heterogeneous molecular defects and phenotypic variability (Reviewed in [3]). Around 60% of patients (ICF1) have mutations in the catalytic domain of the *de novo* DNA methyltransferase (DNMT) DNMT3B [4-6] leading to reduced enzymatic activity [7,8] associated with a significant loss of DNA methylation, notably at juxtacentromeric satellite repeats on chromosome 1 and 16, and less frequently 9 (Reviewed in [9]). The remainder have either non-sense mutations in the zinc-finger and BTB domain-containing 24 (*ZBTB24*) gene (ICF2) [10] or no identifiable mutation in either *DNMT3B* or *ZBTB24* coding sequences (ICFX) [11]; both ICF2 and ICFX show hypomethylation of centromeric alpha-satellites (α-Sat) in addition to the above mentioned repeats [12]. Hypomethylation of satellite repeats is associated with centromeric instability and constitutes an invariant molecular hallmark of ICF patients. Chromosomal anomalies are detectable by karyotype analysis of mitogen-stimulated lymphocytes and that is used to establish the diagnosis [13,14].

Data obtained in ICF lymphoblastoid cell lines (LCLs) showed that DNMT3B mutations also lead to hypomethylation and perturbed expression of several hundred of genes involved in immune function, development and neurogenesis, being both up- and down-regulated, which probably account for the phenotypical manifestations documented in patients [15-17]. Additional molecular mechanisms acting in *trans*, such as perturbed nuclear organization as a result of the altered juxtacentromeric or telomeric heterochromatin organization [18-20], deregulated expression of small regulatory microRNAs (miRNA) [21] or changes in replication timing [22] may also have a deep impact on the deregulation of gene expression programs in ICF patients. However, these perturbed profiles showed great variability, probably reflecting phenotypic variability between patients and cell culture effects, and no common or distinguishing molecular signatures could be reliably established from these studies.

Our analysis of perturbed DNA methylation patterns and expression programs in a mouse model for ICF1 revealed the striking over-representation of germline genes among the most upregulated genes [23]. In addition, our data suggested a new role for Dnmt3b in the protection of somatic cells against the promiscuous expression of the germ line program, playing the central role in both the establishment and maintenance of DNA methylation profiles at these genes [24]. Although the impact of their illicit expression on ICF phenotypes remains to be solved, their inherent repressed state in all somatic cells makes them good candidate biomarkers of molecular dysfunctions in ICF syndrome. We tested hypomethylation and illicit expression of the thus identified germline genes in a cohort of ICF patients that included five newly enrolled patients, in order to establish a molecular signature easily testable directly from the blood of ICF patients.

## Material and methods

This study was conducted in accordance with the Helsinki Declaration, with informed consent obtained from each patient or the patient’s family. The study was approved by the local ethics committee of Necker-Enfants Malades Hospital, Paris, France.

An expanded Methods section for DNA methylation and gene expression analysis can be found in Additional file 1.

### Healthy donors and patients

Blood samples of six healthy volunteers, aged 35, 24, 26, 27, 29 and 41 years, respectively, were collected and were numbered from 1 to 6. Donors 3 and 6 are women.

Our cohort of patients included thirteen ICF1 patients with mutations in *DNMT3B*, six ICF2 patients with mutations in *ZBTB24* and four ICFX patients with as of yet unknown mutations (Additional file 2). Most patients were described earlier [11,12,25-27] except for five newly enrolled patients (Table 1). The ICF B-lymphoblastoid and fibroblasts named here pCor were obtained from the Coriell Cell Repositories (USA) ( http://ccr.coriell.org/). Patients pG, pR, pI, pH, pC, pD, pN, pP, pS were recruited by the ICF Consortium and described together with patients pG, pR, pI, pH, pC, pD, pN, pP, pS in [12]. Patients pW, pT and P5 were described earlier [11,25,26]. Patients P7 and P8 were recently classified as ICF2 patients [27]. Patients pC, pS, pU and pN were classified as ICFX patients since sequence analysis of *DNMT3B* and *ZBTB24* genes performed as previously described [12,27] did not reveal any mutation in their coding sequences.

**Table 1:** Genetic characteristics of newly identified ICF1 patients

**Patients**	**P1**	**P2**	P3^*^	P4^*^	**pY**
**Year of birth**	2010	2008	2009	2013	1996
**Gender**	F	M	F	F	M
**Amino acid changes in DNMT3B** (NP_008823.1)	p. S655L/ ?	p. R104X/p. I721T	p. G583S/p. G583S	p. G583S/p. G583S	p.T775I/p.T775I
**Consanguinity**	NO	NO	YES	YES	YES

M: male; F: female; *siblings.

New ICF syndrome patients P1, P2, P3, P4 and pY were diagnosed according to cytogenetic analysis that revealed typical multiradial chromosome configurations with multiple arms from chromosomes 1 and 16, and clinical features, mainly primary immunodeficiency. For these patients, primary immunodeficiency and hypomethylation at satellite repeats are detailed in Additional files 3 and 4. These patients were classified as ICF1 patients (Necker Hospital, Paris, France) based on mutations found in *DNMT3B* using previously described sequencing methods [12,27]. Written informed consent was obtained from the parents of the patients.

### Primary cells and cell lines

Primary fibroblasts (passage 7 for pG, pW and pT; passage 5 for pR; passage 6 for pI; passage 14 for pC and pP; passage 9 for pCor and pS) and Lymphoblastoid cell lines (LCLs) from healthy donors and ICF patients were cultured in DMEM and RPMI 1640 respectively, supplemented with 15% FCS, glutamine and antibiotics (Invitrogen).

### Methylation-sensitive restriction enzyme-coupled qPCR assay

Genomic DNA (200 ng) was digested at 37°C for 4 h with 10 U of the methylation-sensitive enzyme *AciI*, or *NcoI* (New England Biolabs) which does not have cutting sites in our regions of interest and served to normalize the data. The endonucleases were subsequently inactivated by incubation at 65°C for 20 min. Real-time PCR was carried out using the light cycler-DNA MasterPLUS SYBR Green I mix (Roche) supplemented with 0.5 μM specific primer pairs and with 2 μL of digested DNA. Real-time quantification PCR were run on a light cycler rapid thermal system (LightCycler®480 2.0 Real time PCR system, Roche) with 20 sec of denaturation at 95°C, 20 sec of annealing at 65°C and 20 sec of extension at 72°C for all primers, and analyzed by the comparative CT (∆CT) method according to the formula: methylation (%) = E^(∆CT)^ × 100 where E represents PCR efficiency and ∆CT = CT_sample_ (AciI digest) - CT_sample_ (NcoI digest). Sequences of primers within CpG islands at germline gene promoters are shown in Additional file 1. Each data shown on histograms is the mean result of qPCR analysis on at least three independent experiments performed on at least three independent genomic extractions.

## Results and discussion

### Hypomethylation and expression of *MAEL* and *SYCE1* define molecular markers for ICF1 syndrome

Peripheral blood samples were obtained from healthy donors and ICF patients. We assessed profiles of gene expression focusing on the germline genes transcriptional signature that we previously established in a mouse model for ICF1 [23,24].

**Figure 1:**
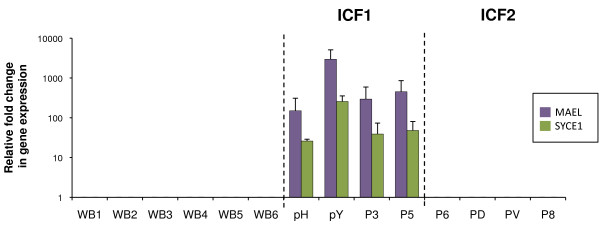
**Expression of *MAEL *and *SYCE1 *in whole blood defines specific biomarkers for ICF1 patients.** Expression levels of *MAEL* (purple bars) and SYCE1 (green bars) were assessed by qRT-PCR, normalized to U6 snRNA levels and presented as fold change relative to that of control whole blood from healthy donors (WB). ICF subtypes 1 and 2 are indicated and separated by dotted lines. Raw data used to built this Figure can be found in Additional file 6. WB, control whole blood from healthy donors. Error bars represent standard error.

As expected from their expression primarily in germ cells (Additional file 5; and RNAseq and exon arrays datasets on normal tissues publically available at UCSC genome browser) and function in meiosis, expression of the germline genes tested was undetectable in blood cells from healthy controls, although that of SYCE1 showed some degree of variation between healthy donors that did not seem to correlate with age or sex (Figure 1 and Additional file 6). In contrast, strong expression was detected for two of them in blood cells from ICF patients; among all the germline genes tested, only that of MAELSTROM (*MAEL*), involved in repression of transposable elements during meiosis, and Synaptonemal Complex Central Element Protein 1 (*SYCE1*), involved in assembly of the synaptonemal complex during meiosis in germ cells, were transcribed in blood cells from ICF patients (Figure 1 and Additional files 6 and 7). This illegitimate expression in whole blood was very strong and remarkably specific for ICF1 patients while at significantly lower levels in ICF2 blood cells (raw data and p values in Additional file 6). Interestingly, patient P5 had only mild ICF phenotype [25] although he showed strong expression of *MAEL* and *SYCE1*. Therefore, given their repressed state in normal somatic tissues, detection of *MAEL* and *SYCE1* expression in blood cells provides a statistically significant signature for the most prevalent sub-type of the disease, regardless of the severity of the symptoms.

**Figure 2:**
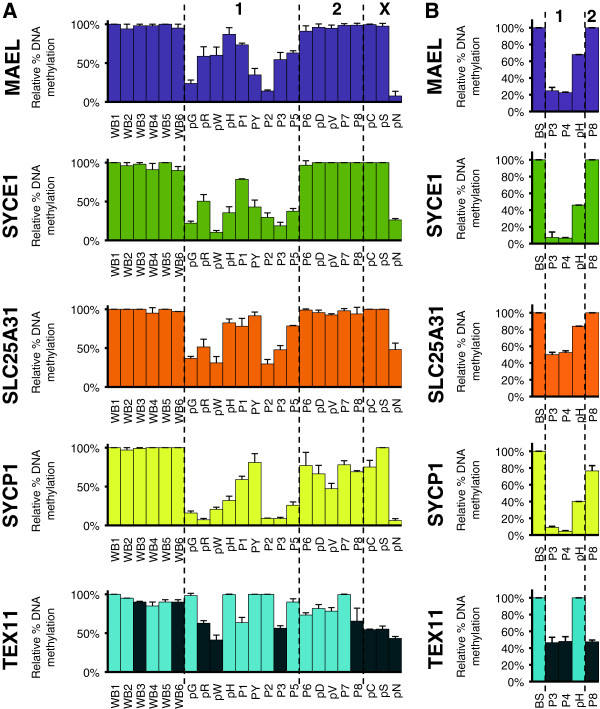
**Relative DNA methylation levels at germline gene promoters in whole blood and buccal swabs from ICF patients.** Methylation analysis in whole blood **(A)** and buccal swabs **(B)** were assessed by Methylation-Sensitive Restriction Assay, followed by qRT-PCR amplification of the AciI digested product with primers flanking at least two AciI sites within the promoter CpG island. A non-cutter NcoI control digest served to normalize data that are presented as a percentage of methylation relative to the control digest. ICF subtypes 1, 2, and X are indicated and separated by dotted lines. For the X-linked gene TEX11, female patients are indicated as black bars. Raw data used to built this Figure can be found in Additional file 8. WB and BS are control whole blood DNA and buccal swabs from healthy donor, respectively. Error bars represent standard error.

Since the germline genes tested in this study are known to be repressed through DNA methylation in mice [23,24,28] we analyzed their methylation status on genomic DNA isolated from fresh whole blood samples (Figure 2A) or epithelial cells obtained from buccal swabs (Figure 2B), from ICF patients and healthy donors. Consistent with their expression only in blood cells from ICF1 patients (Figure 1), analysis of methylation at *MAEL* and *SYCE1* revealed their hypomethylated state specifically in ICF1 patients since ICF2 and ICFX patients (except ICFX patient pN; discussed below) were methylated at values comparable to healthy controls (raw data and p values in Additional file 8). Similarly, Solute Carrier family 25 member 31 (*SLC25A31* also known as *ANT4*), encoding a mitochondrial ADP/ATP carrier essential during spermatogenesis, was significantly hypomethylated only in ICF1 patients (Figure 2 and Additional file 8) although this hypomethylation did not correlate with transcriptional activation (Additional file 6).

Importantly, and as mentioned above, this transcriptional signature could not have been unraveled using classically available cultured patient cells like Epstein-Barr virus-transformed lymphoblastoid cell lines (LCLs) and primary fibroblasts. Indeed, although we could easily detect MAEL and SYCE1 transcripts in these cultured cells, this expression was independent of patient genotype (Additional file 7). We also noticed that the illicit expression of *MAEL* in fibroblasts from ICF1 patients became undetectable beyond passage 9 (Additional file 9), although DNA methylation levels at its promoter remained relatively unchanged, suggesting that extensive culture may limit findings of illegitimately expressed genes. Hypomethylation of DNMT3B-target genes in ICF2 and ICFX cells (Additional file 7), in which *DNMT3B* is not mutated, could result from well-known, direct or indirect, aberrant culture-induced defects of DNA methylation [29]. Furthermore, EBV transformation and cell culture perturb DNA methylation patterns in human LCLs, in particular at satellite repeats of juxtacentromeric regions [30] that are used to diagnose ICF patients. Therefore, like in previously reported transcriptomic studies examining differential gene expression profiles between ICF patients and healthy controls [15,16], our data obtained in LCLs or fibroblasts probably reflected culture-associated defects in DNA methylation and gene expression, which have been superimposed on the intrinsic defects that affect patients, and do not allow the reliable identification of biomarkers with diagnostic value.

In contrast, the analysis that we conducted in uncultured cells from patients provided the first blood-based gene expression test to identify ICF1 syndrome, and the first discriminating transcriptional differences amongst ICF patients. The molecular functions of ZBTB24 remain to be identified, but the finding that the germline genes tested herein are not similarly affected by mutations in *DNMT3B* or *ZBTB24* contradicts earlier suggestion that ZBTB24 is a mere adapter of DNMT3B function [12].

### Hypomethylation signature common to patients with mutations in *DNMT3B* or *ZBTB24*

DNA methylation at the promoters of DEAD (Asp-Glu-Ala-Asp) Box Polypeptide 4 (*DDX4*), the homolog of VASA proteins in Drosophila with key roles in germ cells development, and Testis Expressed 12 (*TEX12*), was similar between patients and controls, suggesting that, in contrast to the mouse, methylation of these genes was not affected in human DNMT3B- or ZBTB24-deficient cells (Additional file 10). Interestingly, the Synaptonemal Complex Protein 1 (*SYCP1*) gene showed significant hypomethylation in all but one ICFX patient with unknown mutation. Similarly, we observed hypomethylation of the X-linked testis-expressed gene 11 (*TEX11*) irrespective of ICF subtype (Figure 2; black bars), but restricted to female ICF cells (raw data and p values in Additional file 8). Whether hypomethylation at *SYCP1* and *TEX11* promoters was a direct consequence of ZBTB24 loss of integrity remains to be seen. However, these data identifies *SYCP1* and *TEX11* as the first unique gene loci affected by *ZBTB24* mutations and contribute to a significant progress in our understanding of the etiology of ICF syndrome. In the case of the X-linked gene *TEX11*, it is likely related to the global hypomethylation of the inactive X-chromosome reported in female ICF1 patients [31,32]. Therefore, the hypomethylation of *SYCP1* and *TEX11* (Figure 2 and Additional file 8) regardless of the ICF sub-type represents the first unifying molecular signature for ICF syndrome and suggests that hypomethylation of the inactive X-chromosome in female patients may represent an invariant feature of ICF syndrome. In addition, these data point to a putative role of ZBTB24 in establishment or maintenance of DNA methylation at the inactive X-chromosome.

### A new molecular heterogeneity among ICF patients

The perturbations that we reported here represent a reliable index of DNMT3B dysfunction in ICF1 patients, with the notable exception of patient pN. Based on our predictions of a molecular signature that varies according to ICF genotype, the germline methylation (Figure 2) and expression (Figure 1) signatures that we found in ICFX patient pN would place this patient in the ICF1 subtype. However, no mutation was previously found in the coding regions of DNMT3B for this patient [12] or in a new sequencing of DNMT3B and ZBTB24 exons. Intriguingly, the ICFX patient pN differs from other ICFX patients in that this patient had some degree of hypomethylation at α-Sat repeats, intermediate between hypomethylation found in ICF2 cells and almost full methylation characteristic of normal and ICF1 cells (Additional file 4). In addition, this profile resembles that of patient P1, first classified as ICF1 because of mutations in DNMT3B maternal allele but for whom no mutation could be found in the coding regions of the paternal allele. The combination of hypomethylation at *MAEL* and *SYCE1* germline genes, indicative of DNMT3B impaired activity, with modest but reproducible hypomethylation at α-Sat repeats, which is never observed in ICF1 patients and was instrumental in suggesting genetic heterogeneity among ICF patients, implies an even more complex genetic etiology for these two patients. In the absence of an identified “culprit gene” for these patients, it remains unclear whether a third locus is implicated in ICF syndrome. Alternatively, these patients could be “compound” heterozygotes for mutations in both *DNMT3B* and *ZBTB24*, though it would have to be outside coding regions since exome sequencing did not reveal any mutation in the exons of any of the two genes. The genetic defects in these patients will be probably highly informative from a mechanistic perspective regarding the establishment and maintenance of DNA methylation patterns. These observations also emphasize that additional molecular markers besides the methylation of α-Sat sequences are required to distinguish various classes of ICF patients.

### Expression and methylation of germline genes to diagnose individuals with suspected ICF syndrome

**Figure 3:**
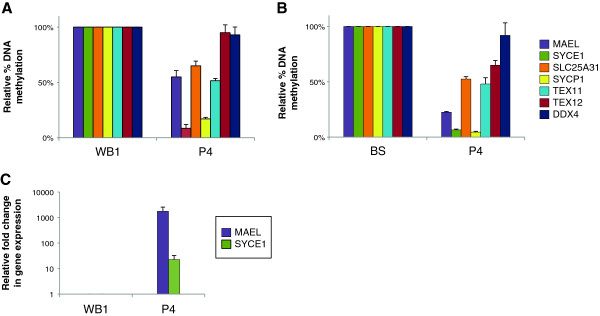
**Hypomethylation coupled to illegitimate expression of *MAEL *and *SYCE1 *to diagnose ICF1 syndrome.** Uncultured cells from a child born to a consanguineous family with one sick children were used to test the power of these biomarkers. Methylation analysis **(A and B)** at germline gene promoters indicated on the right was carried out as in Figure 2, from whole blood **(A)** and buccal swabs **(B)**. Expression analysis of *MAEL* and *SYCE1***(C)**, the only germline genes to be transcriptionally activated upon hypomethylation, was performed from whole blood samples as in Figure 1. The newborn, a sibling of P3, is now patient P4 after the ICF syndrome was confirmed by sequencing of DNMT3B alleles. Raw data used to built this Figure can be found in Additional file 8. WB, Control Whole blood DNA from healthy donor; BS, control buccal swab from healthy donor; Errors bars represent standard error.

We propose that profiling of DNA methylation at *MAEL* and *SYCE1* germline genes, combined to the detection of their illicit expression using non-invasive diagnostic techniques, could serve as a powerful, quick, and simple procedure to diagnose patients with suspected ICF1 syndrome. Because these genes are normally repressed in somatic tissues, any diagnostic test based on their expression will give an unambiguous result. To test the power of these genes for diagnostic means, we assessed methylation and expression profiles of germline genes in a child born to a consanguineous family but whose genotype at the time of testing was not known. This child, who is a sibling of patient P3, was born during the course of this study, and we were able to sample buccal epithelial cells and peripheral blood when she was 3 months old. Our analysis revealed germline gene methylation and transcription profiles typical of that of ICF1 patients tested in this study (Figure 3 and p values in Additional file 8). These observations were highly suggestive of the ICF1 syndrome in this child, who we named patient P4 (Table 1). In line with our predictions, diagnosis of the ICF1 syndrome was subsequently confirmed by sequencing of DNMT3B exons and characterization of the same mutations identified in her older sister (Table 1).

## Conclusions

Correlations between genotype and phenotype of ICF patients are emerging [11], and the defining of a reliable ICF-specific molecular signatures will help to explain such differences in the clinical manifestations of ICF syndrome amongst ICF patient subtypes. In addition to providing a new contribution to the characterization of unifying and distinguishing molecular signatures between ICF patient subtypes, this first molecular signature identifiable in the peripheral blood or epithelial cells of patients could be easily introduced into routine clinical immunology or neurology departments to facilitate testing of patients with suspected ICF syndrome. Because ICF is probably under-diagnosed, we predict that the analysis of such markers will greatly aid diagnosis and prioritize patients for mutation screening in cases where performing exome-sequencing or cytogenetics analysis may represent a challenge or when ICF should be suspected like in patients with immunodeficiency as the presenting factor if associated with facial anomalies [33], in patients that present an incomplete ICF phenotype or individuals from consanguineous families [25], or newborns from families where ICF cases have already been reported (this study). In addition, our analysis allowed the identification of unprecedented molecular characteristics in two ICF patients, suggestive of a novel type of genetic origin for the disease, stressing the need to continue the search for mutations that lead to immunodeficiency associated with chromosomal instability. The hallmark of ICF being hypomethylation of heterochromatin DNA repeats, combined, these efforts will have great impact on our understanding of the DNA methylation pathways.

## Competing interests

The authors declare that they have no competing interest.

## Authors’ contributions

GV, ELW and SH carried out the molecular genetic studies. DS carried out the immunological characterization of newly enrolled ICF patients. HN, IY, HS carried out the exome sequencing of ICFX patients. FF, AM, CP collected the biological samples from ICF patients. GV and CF designed the study. GV coordinated the sample and data collection and analyzed the data. GV and ELW drafted the manuscript. CF supervised the study and revised the manuscript. All authors read and approved the final manuscript.

## Supplementary Material

Additional file 1 **Additional methods section for DNA methylation and gene expression analysis.** Table of primers used for PCR amplification. Click here for file

Additional file 2 Table summarizing genetic mutations found in ICF patients already described and associated references. Click here for file

Additional file 3 Table showing immunological characteristics of newly identified ICF patients. Click here for file

Additional file 4 Southern blot analysis of DNA methylation at centromeric and juxta-centromeric regions of ICF patients. Click here for file

Additional file 5 Germline genes expression and promoter methylation in control conditions including control fibroblasts as somatic cells, human testes and a cell line treated by the demethylating agent 5-azacytidine. Click here for file

Additional file 6 **Raw PCR data used to built expression histograms shown in Figure** 1**, and statistical analysis to compare healthy controls and ICF patients.** Click here for file

Additional file 7 **(A) Additional information on expression analysis performed in cultured cells from patients, EBV-transformed lymphocytes or immortalized fibroblasts, suggesting that these cellular systems cannot provide reliable molecular markers for a disease with methylation defects. (B)** Expression analysis and DNA methylation of germ line genes in lymphoblastoid cell lines from patients. **(C)** Expression analysis and DNA methylation of germ line genes in immortalized fibroblasts from patients. Click here for file

Additional file 8 **Raw PCR data used to built DNA methylation histograms shown in Figure** 2**, and statistical analysis to compare healthy controls and ICF patients.** Click here for file

Additional file 9 Control experiment showing that expression of Maelstrom decreases with the number of passages in culture. Click here for file

Additional file 10 **DNA methylation analysis at *TEX12 *** **and *DDX4 *** **promoters showing that their dependency on DNMT3B for methylation and silencing in murine cells is not conserved in humans.** Click here for file
