# Supplementary material for: Germline genes hypomethylation and expression define a molecular signature in peripheral blood of ICF patients: implications for diagnosis and etiology
Source: Orphanet J Rare Dis. 2014 Apr 17;9:56. doi: 10.1186/1750-1172-9-56 (PMC4022050; doi:10.1186/1750-1172-9-56)
Supplement: Additional file 1 — Additional methods section for DNA methylation and gene expression analysis. Table of primers used for PCR amplification. [file 1750-1172-9-56-S1.pdf]

ADDITIONAL FILE 1

Preparation of RNA and genomic DNA

Total RNA from cell lines and whole blood was isolated using TRIzol® Reagent and TRIzol® LS Reagent (Invitrogen), respectively, according to manufacturer's instructions. Contaminant genomic DNA was eliminated with TURBO DNA-free kit (Ambion).

Genomic DNA was extracted and purified from cell lines, whole blood or buccal swab using the NucleoSpin®Tissue kit (Macherey-Nagel) according to manufacturer's instructions. The DNA pellet was eluted in TE containing 20 μg/mL RNAse A.

Methylation analysis by Southern blotting

Genomic DNA from fresh blood (500 ng) was digested with 20 units of BstBI or HhaI (New England Biolabs) for 16h to analyze the DNA methylation patterns of Sat II and α-Sat repeats, respectively. The digested DNA fragments were separated by electrophoresis using 1% agarose gels and transferred overnight to Hybond-N^+ membranes (GE Healthcare) in 20X SSC. After a UV-crosslink, the membranes were pre-hybridized in 6X SSC, 5X Denhardt and 0.1% SDS and then hybridized with ^{32}P-labeled Sat II or α-Sat oligonucleotide probes (5'-TCGAGTCCATTCGATGAT-3' or 5'-ATGTGTGCATTCAACTCACAGAGTTGAAC-3'). Pre-hybridization and hybridization was carried out at 42°C for 1h. The membranes were washed 3 times in 6X SSC and 0.1%SDS at 37°C and then subjected to phosphorimaging using FLA 7000 phosphorimager (Fuji).

Analysis of gene expression

Reverse transcription was carried out using 1 μg DNA-free RNA and 50 μM random hexamers, 20U of RNase Out and 100U of Superscript III reverse transcriptase (Invitrogen). Complementary DNA

reactions were used as templates for PCR reactions. Real-time PCR was performed using the light cycler-DNA MasterPLUS SYBR Green I mix (Roche) supplemented with 0.5 μM specific primer pairs (**Table below**). Real-time quantitative PCR were run on a light cycler rapid thermal system (LightCycler®480 2.0 Real time PCR system, Roche) with 20 sec of denaturation at 95°C, 20 sec of annealing at 60°C and 20 sec of extension at 72°C for all primers, and analyzed by the comparative CT (ΔCT) method. Each data shown in qPCR analysis is the result of at least three independent experiments performed on at least three independent RNA extraction.

Primers used in Methylation-Sensitive Restriction Enzyme-Coupled qPCR assay and qRT-PCR

Gene	NCBI Reference Sequence	Primers MSRE	Primers qRT-PCR
**MAEL**<br>1q24.1	NM_032858	5'-CCAGCCAATCAGAGCACTTG-3'<br>5'-GCTCCCGCCCTAAGTAACAG-3'	5'-AAGGCCAGCCGGAATGCTTACTATT-3'<br>5'-CTCCATTCTCGAGCCATTTCTGCGT-3'
**SYCE1**<br>10q26.3	NM_001143763	5'-GGCAGGCTACGCTCCTCTG-3'<br>5'-GCGAGTGAAATCTGCGGCAG-3'	5'-GCAGAAGGAACTGGACTCGC-3'<br>5'-CCTGCAACATGGTGTGCTTC-3'
**DDX4**<br>5p15.2-p13.1	NM_001166533	5'-AGGGAATCCGCAGGCTAGA-3'<br>5'-CAGTTCCCGTAGAAAAGCGG-3'	5'-ATGTGCTACTCCTGGAAGACTG-3'<br>5'-CCAACATGCGATCAGCTTCATC-3'
**TEX12**<br>11q22	NM_031275	5'-GGTGGTGGTGGGAGAGCTG-3'<br>5'-CAGTCCGTCACATTCTCACAGG-3'	5'-GAGTCTCCAGTGCCAGATAGTC-3'<br>5'-GCATCTACTGCTGCTCTCTCAC-3'
**SYCP1**<br>1p13-p12	NM_003176	5'-GCACAGAACCCACGGTTTCC-3'<br>5'-GCAGTAGCACAACATCGCCC-3'	5'-GAAGGAAACGAGGGTTTATTCC-3'<br>5'-CAAAGGGCTTTTGCTTTTCCAT-3'
**TEX11**<br>Xq13.1	NM_031276	5'-ACTCTCTGAACTGCATCCCCA-3'<br>5'-CCACTAAACCACTCACCTCA-3'	5'-GAATCTGTTGGGTTTCATTTCCTG-3'<br>5'-AACCAGTTCATTGATTCTGCTGTC-3'
**SLC25A31**<br>4q28.1	NM_031291	5'-GCCACTTTCTCGCCAGTACGA-3'<br>5'-GTACGGGAGCTACGATGAAGGG-3'	5'-GGGGCAACATCCTTATGTGT-3'<br>5'-GTACTGAAACACCAAACCCT-3'
