# Supplementary material for: Germline genes hypomethylation and expression define a molecular signature in peripheral blood of ICF patients: implications for diagnosis and etiology
Source: Orphanet J Rare Dis. 2014 Apr 17;9:56. doi: 10.1186/1750-1172-9-56 (PMC4022050; doi:10.1186/1750-1172-9-56)
Supplement: Additional file 2 — Table summarizing genetic mutations found in ICF patients already described and associated references. [file 1750-1172-9-56-S2.pdf]

Additional File 2. Genetic status of ICF patients.

Patients (Gender)	Amino acid changes in DNMT3B	Amino acid changes in ZBTB24	Birth year	Follow-up Status (reference)
**ICF1**	NP_008823.1			
**pG (M)**	p. I41fsX42 / p.S780L		1985	Deceased age 16 [1]
**Cor (F)**	p. A603T / p.R807delinsSTP			NA [2]
**pR (F)**	p. D817G / p. D817G		1991	Necker Hospital (Paris, France) [3]
**pW (F)**	p.V613A / p.R826C		2004	NA [4]
**pT (F)**	p. D817G / p. D817G		2004	Deceased [5]
**pI (M)**	p. G663S / p.V726G		1972	Deceased [6]
**pH (M)**	p.V818M / p.V818M		1990	University Hospital Center (Nancy, France) [3]
**P1*(F)**	p. S655L / ?		2010	Necker Hospital (Paris, France)
**pY *(M)**	p.T775I / p.T775I		1996	Necker Hospital (Paris, France)
**P2* (M)**	p. R104X / p.I721T		2008	Necker Hospital (Paris, France)
**P3[a]* (F)**	p. G583S / p. G583S		2009	Necker Hospital (Paris, France)
**P4[a]* (F)**	p. G583S / p. G583S		2013	Necker Hospital (Paris, France)
**P5 (M)**	p. K770E / p. K770E		1991	(Lebanon) [7]
**ICF2**		**NP_055612.2**		
**pP (F)**		p.R457X / p.R457X	1981	NA [8]
**P6[b] (M)**		p.H132Q fsX19 / p.H132Q fsX19	2003	Hotel-Dieu de France Hospital (Beirut, Lebanon) [9]
**pD[b] (M)**		p.H132Q fsX19 / p.H132Q fsX19	1997	Hotel-Dieu de France Hospital (Beirut, Lebanon) [9]
**pV[b] (M)**		p.H132Q fsX19 / p.H132Q fsX19	1998	Hotel-Dieu de France Hospital (Beirut, Lebanon) [9]
**P7 (M)**		p.R320X / p.R320X	1971	Deceased [10]
**P8 (F)**		p.K263X / p.C327W fsX54	1996	Necker Hospital (Paris, France) [10]
**ICFX**				
**pC (F)**				NA [3]
**pS (F)**			1978	Necker Hospital (Paris, France) [3]
**pU (M)**				NA
**pN (F)**				NA [3]

M = male ; F = female ; * and grey shade = newly described ICF1 patients in the current study ; [a ,b] siblings**;** NA; not available

REFERENCES

1. Turleau C, Cabanis MO, Girault D, Ledeist F, Mettey R, Puissant H, Prieur M, de Grouchy J: Multibranched chromosomes in the ICF syndrome: immunodeficiency, centromeric instability, and facial anomalies. *Am J Med Genet* 1989, 32:420-424.
2. Carpenter NJ, Filipovich A, Blaese RM, Carey TL, Berkel AI: Variable immunodeficiency with abnormal condensation of the heterochromatin of chromosomes 1, 9, and 16. *J Pediatr* 1988, 112:757-760.
3. Jiang YL, Rigolet M, Bourc'his D, Nigon F, Bokesoy I, Fryns JP, Hulten M, Jonveaux P, Maraschio P, Megarbane A, et al: DNMT3B mutations and DNA methylation defect define two types of ICF syndrome. *Hum Mutat* 2005, 25:56-63.
4. Weemaes CM, van Tol MJ, Wang J, van Ostaijen-Ten Dam MM, van Eggermond MC, Thijssen PE, Aytekin C, Brunetti-Pierri N, van der Burg M, Graham Davies E, et al: Heterogeneous clinical presentation in ICF syndrome: correlation with underlying gene defects. *Eur J Hum Genet* 2013.
5. Rigolet M, Gregoire A, Lefort G, Blanchet P, Courbes C, Rodiere M, Sarda P, Viegas-Pequignot E: Early prenatal diagnosis of ICF syndrome by mutation detection. *Prenat Diagn* 2007, 27:1075-1078.
6. Hulten M: Selective somatic pairing and fragility at 1q12 in a boy with common variable immunodeficiency. *Clin Genet* 2008, 14:294-295.
7. Brun ME, Lana E, Rivals I, Lefranc G, Sarda P, Claustres M, Megarbane A, De Sario A: Heterochromatic genes undergo epigenetic changes and escape silencing in immunodeficiency, centromeric instability, facial anomalies (ICF) syndrome. *PLoS One* 2011, 6:e19464.
8. Maraschio P, Zuffardi O, Dalla Fior T, Tiepolo L: Immunodeficiency, centromeric heterochromatin instability of chromosomes 1, 9, and 16, and facial anomalies: the ICF syndrome. *J Med Genet* 1988, 25:173-180.
9. Chouery E, ACorbani S, el Ali N, Korban R, Salem N, Castro C, Klayme S, Azoury-Abou Rjeily M, Khoury-Matar R, Debo G, et al: A novel deletion in ZBTB24 in a Lebanese family with immunodeficiency, centromeric instability, and facial anomalies syndrome type 2. *Clin Genet* 2012, 82:489-493.
10. Nitta H, Unoki M, Ichiyanagi K, Kosho T, Shigemura T, Takahashi H, Velasco G, Francastel C, Picard C, Kubota T, Sasaki H: Three novel ZBTB24 mutations identified in Japanese and Cape Verdean type 2 ICF syndrome patients. *J Hum Genet* 2013.
