# Supplementary material for: Germline genes hypomethylation and expression define a molecular signature in peripheral blood of ICF patients: implications for diagnosis and etiology
Source: Orphanet J Rare Dis. 2014 Apr 17;9:56. doi: 10.1186/1750-1172-9-56 (PMC4022050; doi:10.1186/1750-1172-9-56)
Supplement: Additional file 3 — Table showing immunological characteristics of newly identified ICF patients. [file 1750-1172-9-56-S3.pdf]

Patients	P1	P2	P3	P4	pY
**Age**	8 months	6 months	1.5 years	2 months	5 years
**T Lymphocytes**<br>CD4<br>CD8<br>% Naive CD4<br>% Naive CD8	1,133 *(1,400-11,500)*<br>177 *(1,000-7,200)*<br>942 *(200-5,400)*<br>26 *(77-97)*<br>4 *(31-100)*	3,498 *(1,400-11,500)*<br>2,226 *(1,000-7,200)*<br>954 *(200-5,400)*<br>49 *(77-97)*<br>46 *(31-100)*	2,432 *(700-8,800)*<br>1,952 *(400-7,200)*<br>384 *(200-2,800)*<br>ND<br>ND	2,336 *(2200-9,200)*<br>1,440 *(1600-6,500)*<br>672 *(300-3,400)*<br>50 *(73-100)*<br>75 *(47-100)*	1,650 *(850-4,300)*<br>1,034 *(500-2,700)*<br>594 *(9-49)*<br>40 *(52-92)*<br>86 *(19-100)*
**T cell proliferation**<br>PHA	Normal	Normal	ND	Normal	Normal
**B Lymphocytes**<br>CD19+<br>%CD19+ CD27+	250 *(130-6,300)*<br>1 *(4.3-8.3)*	689 *(130-6,300)*<br>2.6 (4.3-8.3)	512 *(160-3,700)*<br>1 (7.5-10.9)	640 (520-2,300)<br>1.3 (2.9-4.5)	308 (180-1,300)<br>1 (11.1-20.4)
**Immunoglobulins**<br>IgG<br>IgA<br>IgM	3.79 *(3.35-6.23)*<br><0.06 *(0.27-0.86)*<br><0.06 *(0.48-1.36)*	2.9 *(2.35-4.37)*<br><0.04 *(0.20-0.62)*<br>0.67 *(0.34-0.95)*	2.87 *(4.82-8.96)*<br><0.05 *(0.33-1.22)*<br><0.05 *(0.5-1.53)*	1.3 *(2.95-5.49)*<br>0.21 *(0.12-0.38)*<br><0.1 *(0.30-0.85)*	0.57 *(5.49-10.19)*<br><0.06 *(0.41-1.41)*<br><0.04 *(0.54-1.53)*

Additional File 3. Immunological status of newly described ICF patients. Lymphocyte counts (/μl) and serum immunoglobulins (g/l) are indicated. Reference intervals for age are indicated into brackets.
