# Supplementary material for: Germline genes hypomethylation and expression define a molecular signature in peripheral blood of ICF patients: implications for diagnosis and etiology
Source: Orphanet J Rare Dis. 2014 Apr 17;9:56. doi: 10.1186/1750-1172-9-56 (PMC4022050; doi:10.1186/1750-1172-9-56)
Supplement: Additional file 4 — Southern blot analysis of DNA methylation at centromeric and juxta-centromeric regions of ICF patients. [file 1750-1172-9-56-S4.pdf]

Velasco et al., Additional File 4

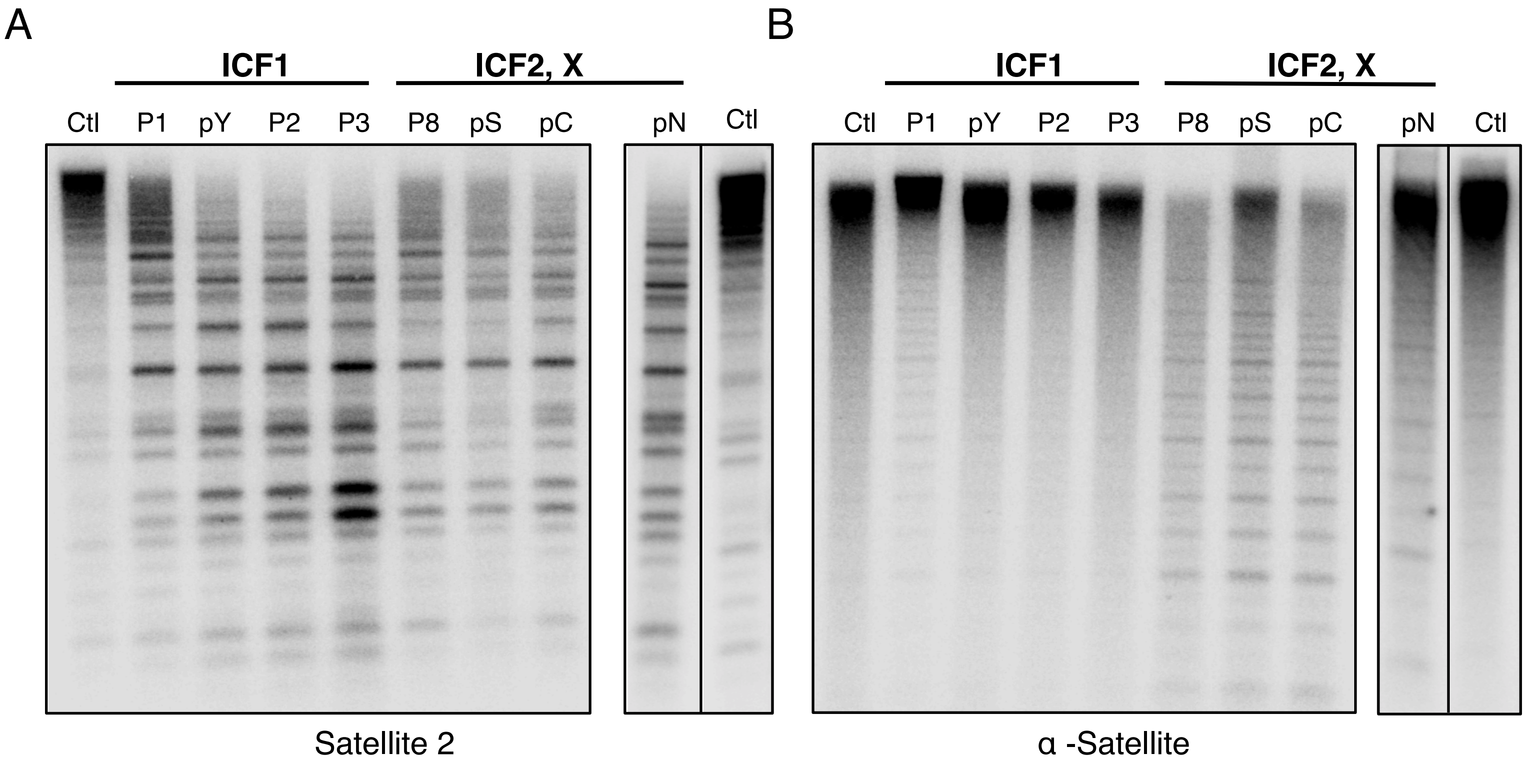

Additional File 4. DNA methylation patterns at Satellite 2 and α-Satellite repeats in whole blood from ICF patients. Genomic DNA from control blood (Ctl), ICF1 patients (P1, pY, P2, P3), ICF2 patient (P8) or ICFX patients (pS and pC) was digested by methylation-sensitive enzymes BstBI (A) or HhaI (B) and subjected to Southern blot analysis using radiolabeled probes complementary to Satellite 2 (A) or α-Satellite (B) repeats. Thin black lines separate lanes that were run on the same gel but were not contiguous (right panels, samples pN and its control lane).
