# Supplementary material for: Germline genes hypomethylation and expression define a molecular signature in peripheral blood of ICF patients: implications for diagnosis and etiology
Source: Orphanet J Rare Dis. 2014 Apr 17;9:56. doi: 10.1186/1750-1172-9-56 (PMC4022050; doi:10.1186/1750-1172-9-56)
Supplement: Additional file 5 — Germline genes expression and promoter methylation in control conditions including control fibroblasts as somatic cells, human testes and a cell line treated by the demethylating agent 5-azacytidine. [file 1750-1172-9-56-S5.pdf]

Velasco et al., Additional File 5

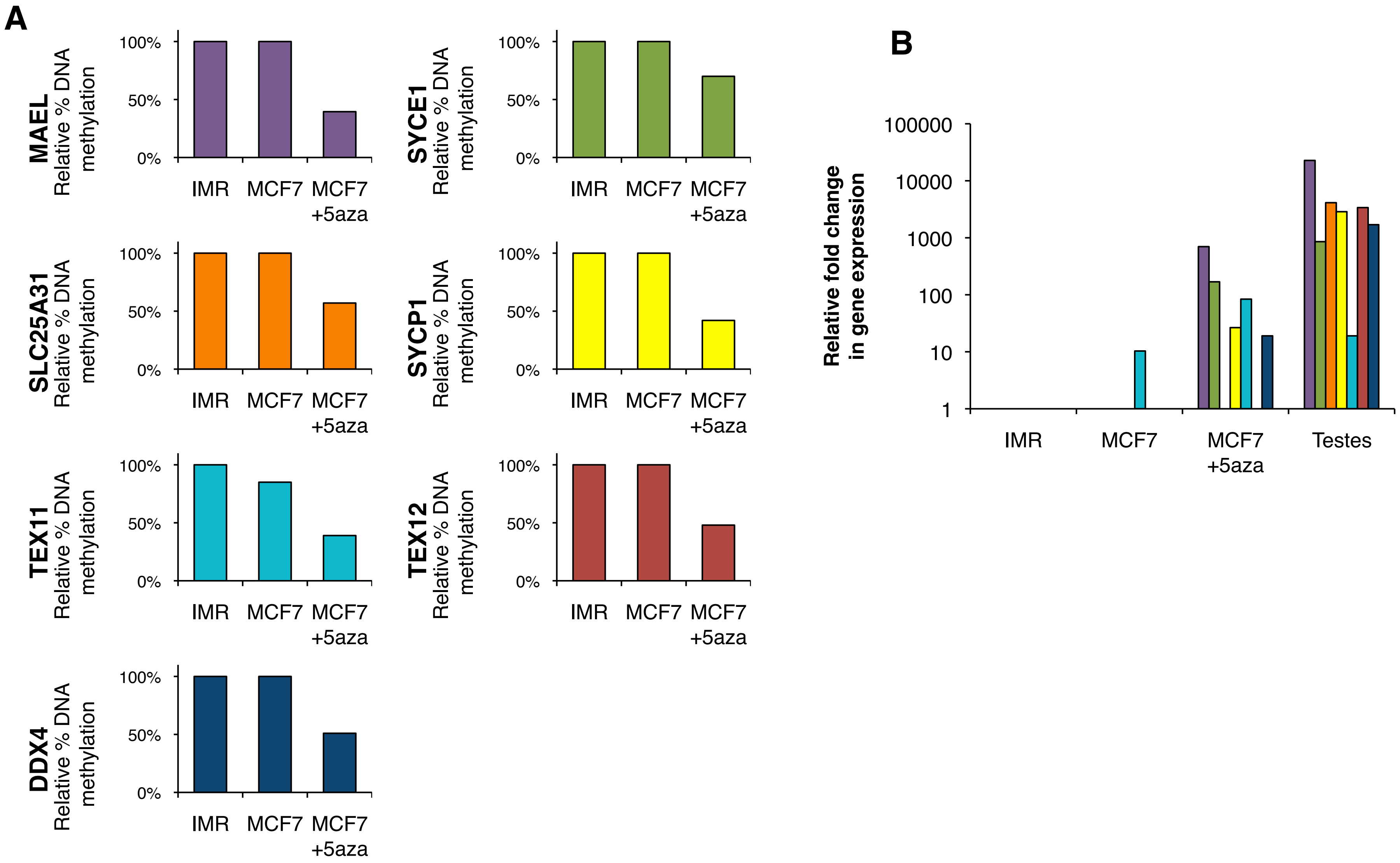

Additional File 5. Analysis of promoter methylation and expression of germline genes in control conditions. (A) DNA methylation was assessed as in Figure 2. (B) Expression of germline genes was assessed by qRT-PCR as in Figure 1, normalized to U6 snRNA levels and presented as fold change relative to the primary human fibroblast line IMR-90. The breast cancer cell line MCF7 treated by the demethylating agent 5- azacytidine (5aza) and human testes were used as positive controls for DNA methylation and expression assays. Data are expressed as relative changes compared to a negative control, IMR-90 fibroblasts (IMR), where germline genes are repressed by DNA methylation.
