# Supplementary material for: Germline genes hypomethylation and expression define a molecular signature in peripheral blood of ICF patients: implications for diagnosis and etiology
Source: Orphanet J Rare Dis. 2014 Apr 17;9:56. doi: 10.1186/1750-1172-9-56 (PMC4022050; doi:10.1186/1750-1172-9-56)
Supplement: Additional file 6 — Raw PCR data used to built expression histograms shown in Figure 1, and statistical analysis to compare healthy controls and ICF patients. [file 1750-1172-9-56-S6.pdf]

<table>
<tr><th colspan="4">Expression in control blood : ΔCTm (CTm)</th></tr>
<tr><th>Sample</th><th colspan="2">MAEL</th><th>SYCE1</th></tr>
<tr><td>WB1</td><td colspan="2">18.45 (>40)</td><td>12.25 (33.8)</td></tr>
<tr><td>WB2</td><td colspan="2">16.85 (>40)</td><td>10.62 (33.77)</td></tr>
<tr><td>WB3</td><td colspan="2">20.05 (>40)</td><td>15.48 (35.43)</td></tr>
<tr><td>WB4</td><td colspan="2">18.33 (>40)</td><td>10.82 (32.49)</td></tr>
<tr><td>WB5</td><td colspan="2">17.25 (>40)</td><td>15.07 (37.82)</td></tr>
<tr><td>WB6</td><td colspan="2">18.52 (>40)</td><td>18.23 (39.71)</td></tr>
<tr><th colspan="4">Median ΔCTm</th></tr>
<tr><td>CTL</td><td colspan="2">18.39</td><td>13.66</td></tr>
<tr><th colspan="4">Standard error</th></tr>
<tr><td>CTL</td><td colspan="2">1.12</td><td>3.01</td></tr>
<tr><th colspan="4">Expression in ICF1 patients : ΔCTm (CTm)</th></tr>
<tr><td>pH</td><td colspan="2">11 (32.5)</td><td>7.39 (28.88)</td></tr>
<tr><td>pY</td><td colspan="2">8.14 (30.88)</td><td>4.66 (27.39)</td></tr>
<tr><td>P3</td><td colspan="2">9.97 (30.65)</td><td>7.55 (28.24)</td></tr>
<tr><td>P4</td><td colspan="2">9.46 (29.26)</td><td>9.4 (29.20)</td></tr>
<tr><td>P5</td><td colspan="2">11.1 (0.06)</td><td>7.95 (28.44)</td></tr>
<tr><th colspan="4">Median ΔCTm</th></tr>
<tr><td>ICF1</td><td colspan="2">9.97</td><td>7.55</td></tr>
<tr><th colspan="4">Standard error</th></tr>
<tr><td>ICF1</td><td colspan="2">1.22</td><td>1.72</td></tr>
<tr><th colspan="4">p values</th></tr>
<tr><th colspan="2">ICF1 vs CTL</th><th colspan="2">ICF1 vs CTL</th></tr>
<tr><td colspan="2">9.17E-07</td><td colspan="2">2.45E-03</td></tr>
<tr><th colspan="4">ICF2 ΔCTm (CTm)</th></tr>
<tr><td>P6</td><td colspan="2">19.23 (>40)</td><td>11.1 (31.87)</td></tr>
<tr><td>pD</td><td colspan="2">20.13 (>40)</td><td>13.14 (33.01)</td></tr>
<tr><td>pV</td><td colspan="2">20.19 (>40)</td><td>12.15 (31.96)</td></tr>
<tr><td>P8</td><td colspan="2">18.57 (>40)</td><td>11.22 (32.65)</td></tr>
<tr><th colspan="4">Median ΔCTm</th></tr>
<tr><td>ICF2</td><td colspan="2">19.68</td><td>11.69</td></tr>
<tr><th colspan="4">Standard error</th></tr>
<tr><td>ICF2</td><td colspan="2">0.77</td><td>0.95</td></tr>
<tr><th colspan="4">p values</th></tr>
<tr><th>ICF1 vs ICF2</th><th>ICF2 vs CTL</th><th>ICF1 vs ICF2</th><th>ICF2 vs CTL</th></tr>
<tr><td>2.74E-06</td><td>0.08</td><td>2.29E-03</td><td>0.28</td></tr>
</table>

Additional File 6. Analysis of *MAEL* and *SYCE1* gene expression in whole blood. Total RNA was extracted from whole blood from healthy donors (CTL), ICF1 and ICF2 patients and subjected to RT-qPCR using primers specific for MAEL or SYCE1 mRNA. For each sample, the differences in cycle threshold (DCT) between germline gene and control U6 snRNA are indicated as a mean value of 3 independent experiments ($\Delta CTm = CTm_{GENE} - CTm_{U6}$). A mean value of the raw cycle threshold (CTm) values are indicated into brackets. For each population, median and standard error are indicated. For both genes, the differences in expression between ICF1 and CTL or ICF2 blood samples are strongly significant ($p < 0.01$; p value calculated using paired, two-tailed Student's *t*-test) and are highlighted in blue. The values for *MAEL* and *SYCE1* expression in whole blood from the young child diagnosed in Figure 3 are indicated in red.
