# Supplementary material for: Germline genes hypomethylation and expression define a molecular signature in peripheral blood of ICF patients: implications for diagnosis and etiology
Source: Orphanet J Rare Dis. 2014 Apr 17;9:56. doi: 10.1186/1750-1172-9-56 (PMC4022050; doi:10.1186/1750-1172-9-56)
Supplement: Additional file 7 — (A) Additional information on expression analysis performed in cultured cells from patients, EBV-transformed lymphocytes or immortalized fibroblasts, suggesting that these cellular systems cannot provide reliable molecular markers for a disease with methylation defects. (B) Expression analysis and DNA methylation of germ line genes in lymphoblastoid cell lines from patients. (C) Expression analysis and DNA methylation of germ line genes in immortalized fibroblasts from patients. [file 1750-1172-9-56-S7.pdf]

ADDITIONAL FILE 7 (A)

Characterization of newly identified patients

Our cohort of patients included 13 ICF1 patients with mutations in DNMT3B, 6 ICF2 patients with mutations in ZBTB24 and 4 ICFX patients with as of yet unknown mutations. Most of these patients have been described earlier (**Additional File 2**) except for 5 newly enrolled patients (**Table1**).

Humoral immunity in all patients is characterized by hypogammaglobulinemia compared to reference intervals (**Additional File 3**). Absolute and relatives numbers of CD3, CD4 and CD8 T lymphocytes were normal in all but one patient (P1). This patient had a major decrease in $CD4^+$ T cells. $CD45RA^+$ $CD31^+$ naïve T cells were reduced in the $CD4^+$ compartment for all patients whereas naïve T cells were normal in the $CD8^+$ subset for the three patients tested. In four patients tested, normal T-cell proliferation was observed in response to phytohemagglutinin (PHA) mitogen stimulation. Absolute and relative numbers of total B cells were within the normal range for age. However, they all lacked $CD19^+$ $CD27^+$ memory B cells. Vaccine-specific serologies were available for three patients. Specific antibodies were not detected whereas the patients were correctly vaccinated.

Search for mutations showed that these patients can be classified as ICF1 since they hold mutations in *DNMT3B* (**Table 1**). Patients pY, P3 and P4 belong to consanguineous families and are homozygotes for mutations in the catalytic domain of *DNMT3B;* P2 is a compound heterozygote for a mutation in the catalytic domain and a non-sense mutation at the N-terminus, and patient P1 possesses a mutation in the catalytic domain on the maternal allele whereas no mutation could be found within the coding regions of the paternal *DNMT3B* allele nor in *ZBTB24* alleles.

We verified methylation status at satellite repeats, the only molecular signature that categorizes ICF patients in different ICF subtypes, using Southern blotting after digestion of genomic DNA isolated from peripheral blood of these patients with methylation-sensitive restriction enzymes. In accordance

with their classification as ICF1, patient P2, pY, and P3 were hypomethylated on Sat II sequences characterized by the appearance of low molecular weight bands after digestion, but not on α-Sat sequences that were resistant to digestion. As controls, ICF2 patient P8 and ICFX pS and pC showed hypomethylation of both Sat II and α-Sat sequences (**Additional File 4**). The case of patient P1, classified as ICF1 because of a mutation in the DNMT3B maternal allele, is intriguing since he showed a small but appreciable and reproducible degree of hypomethylation at α-Sat repeats.

MAEL and SYCE1 expression can distinguish different sub-types of ICF patients in cultured cells

To address the existence of a molecular signature involving the hypomethylation and illegitimate expression of germline genes in the somatic tissues of ICF patients, we examined the profiles of DNA methylation by Methylation-Sensitive Restriction Enzyme-Coupled qPCR assay (MSRE) and expression by qRT-PCR, focusing on seven germline genes (*MAEL*, *SYCE1*, *SYCP1*, *SLC25A31*, *TEX11*, *TEX12*, and *DDX4*) previously identified as direct targets of Dnmt3b in the mouse [1, 2]. We first used available cultured patient cells, typically Epstein-Barr virus (EBV) transformed lymphoblastoid cell lines (LCLs) and primary fibroblasts. As controls where germline genes are normally silent or expressed, we used RNA extracted from primary IMR-90 fibroblasts, the breast cancer cell line MCF-7 treated or not with the demethylating agent 5-azacytidine or from human testes, respectively (**Additional File 5**).

In contrast to what we observed in the somatic tissues of ICF mice, the majority of the germline genes tested was not illegitimately expressed in ICF patient cells, although all genes showed some degree of perturbation to promoter methylation (**Figures 7B and 7C below**). The differential promoter methylation and expression of the germline genes tested amongst ICF patient subtypes and controls allowed the grouping of these genes into four classes as outlined below:

1. TEX12 and *DDX4* showed varying degrees of reduction in promoter methylation that could not be correlated to ICF subtype, and showed a high degree of variation between LCLs and fibroblasts.

2. TEX11 was hypomethylated in primary fibroblasts of female ICF patients only. This sex-dependent methylation is consistent with the location of this gene on the X chromosome and is likely to reflect previously reported hypomethylation on regions of the inactive X chromosome [3] (changer réf, mettre Miniou et al., 1994). The fact that this pattern was not conserved in LCLs could reflect aberrant methylation associated with EBV transformation or prolonged cell culture.

3. SYCP1 and *SLC25A31* were hypomethylated predominantly in ICF1 patient fibroblasts. In LCLs, a similar profile was found for *SCP1* but not for *Slc25a31* which showed methylation profiles similar to that of control LCLs.

4. MAEL and *SYCE1* were the only two genes to be illegitimately expressed in ICF patient LCLs and fibroblasts (**Figures 7B and 7C**). *MAEL* seemed to be hypomethylated in all ICF patient subtypes. However, its illegitimate activation was specific to ICF1 patients, excluding the ICFX patient pN, for whom hypomethylation and expression could be correlated. *SYCE1* was similarly hypomethylated and expressed in a tissue-independent manner in all ICF1 patients tested. In a handful of ICF2 and ICFX patient *Syce1* was hypomethylated and expressed, albeit at reduced levels compared to ICF1 patients (**Figures 7B and 7C**).

In addition to the low degree of correlation between hypomethylation and expression that we observed in cultured cells (in **Figures 7B and 7C**), we also noted several examples of flagrant differences in methylation between LCLs and primary fibroblasts derived from the same patient at each germline gene tested (**Figures 7B and 7C**; for example, patient pC at *Syce1*). Although this could result from cell-type differences, we also found that hypomethylation of X-linked gene *TEX11* in female patients was more obvious in primary fibroblasts (patients pCor, pR, pW, pT, pP, pC and pS) than in LCLs (patients pCor, pC and pN).

REFERENCES

1. Velasco G, Hube F, Rollin J, Neuillet D, Philippe C, Bouzinba-Segard H, Galvani A, Viegas-Pequignot E, Francastel C: Dnmt3b recruitment through E2F6 transcriptional repressor mediates germ-line gene silencing in murine somatic tissues. *Proc Natl Acad Sci U S A* 2010, 107:9281-9286.
2. Walton EL, Francastel C, Velasco G: Maintenance of DNA methylation: Dnmt3b joins the dance. *Epigenetics* 2011, 6:1373-1377.
3. Hansen RS, Wijmenga C, Luo P, Stanek AM, Canfield TK, Weemaes CM, Gartler SM: The DNMT3B DNA methyltransferase gene is mutated in the ICF immunodeficiency syndrome. *Proc Natl Acad Sci U S A* 1999, 96:14412-14417.

Velasco et al., Additional File 7B

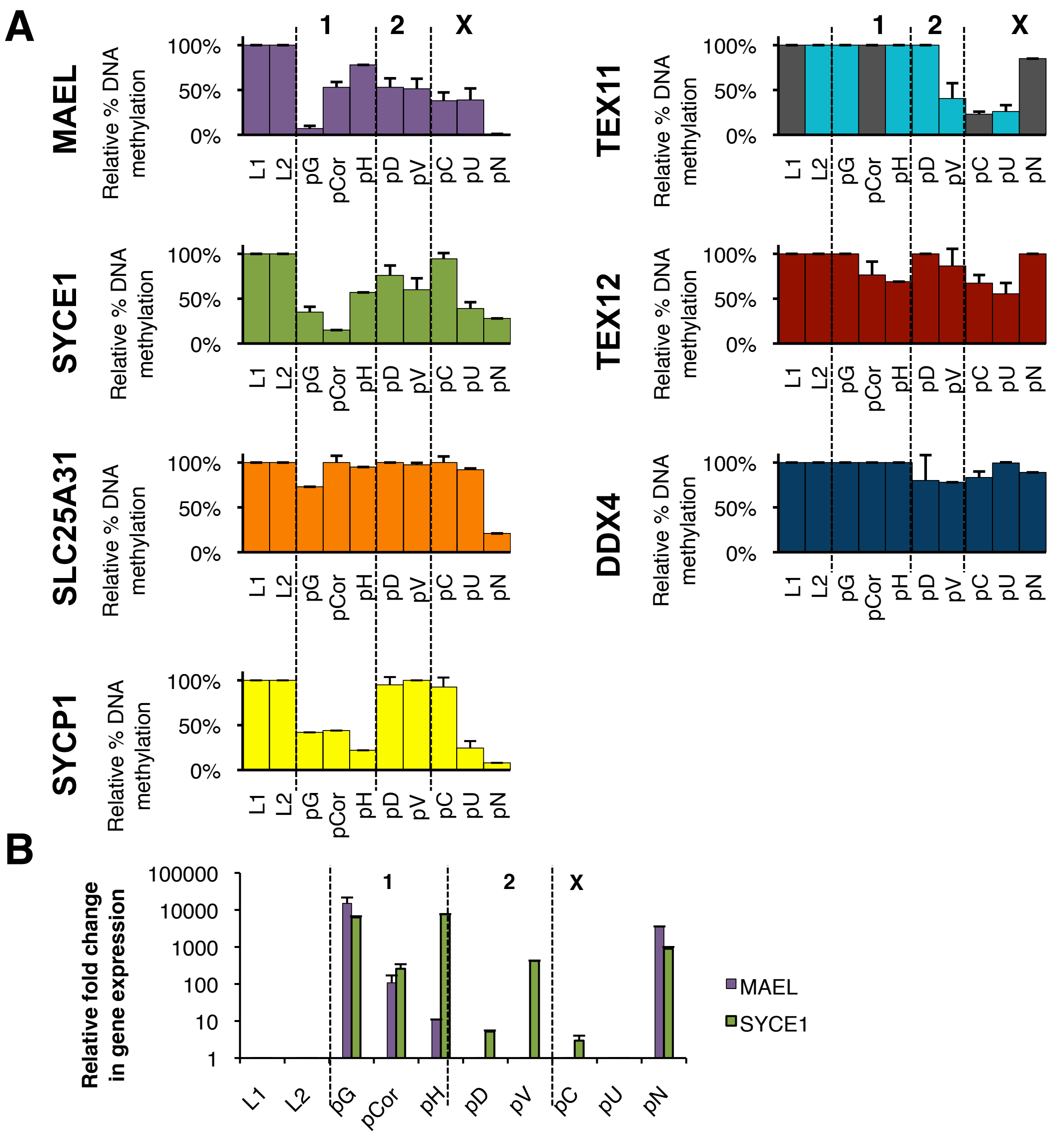

Additional File 7B. Relative promoter methylation and expression analysis of germline genes in patient-derived LCLs. (A) DNA methylation was assessed as in Figure 2. For the X-linked gene TEX11, female patients are indicated as black bars. (B) Expression levels of MAEL (purple bars) and SYCE1 (green bars) were assessed by qRT-PCR, normalized to U6 snRNA levels and presented as fold change relative to that of control LCLs from healthy donors (L1 and L2). Error bars represent standard error.

Velasco et al., Additional File 7C

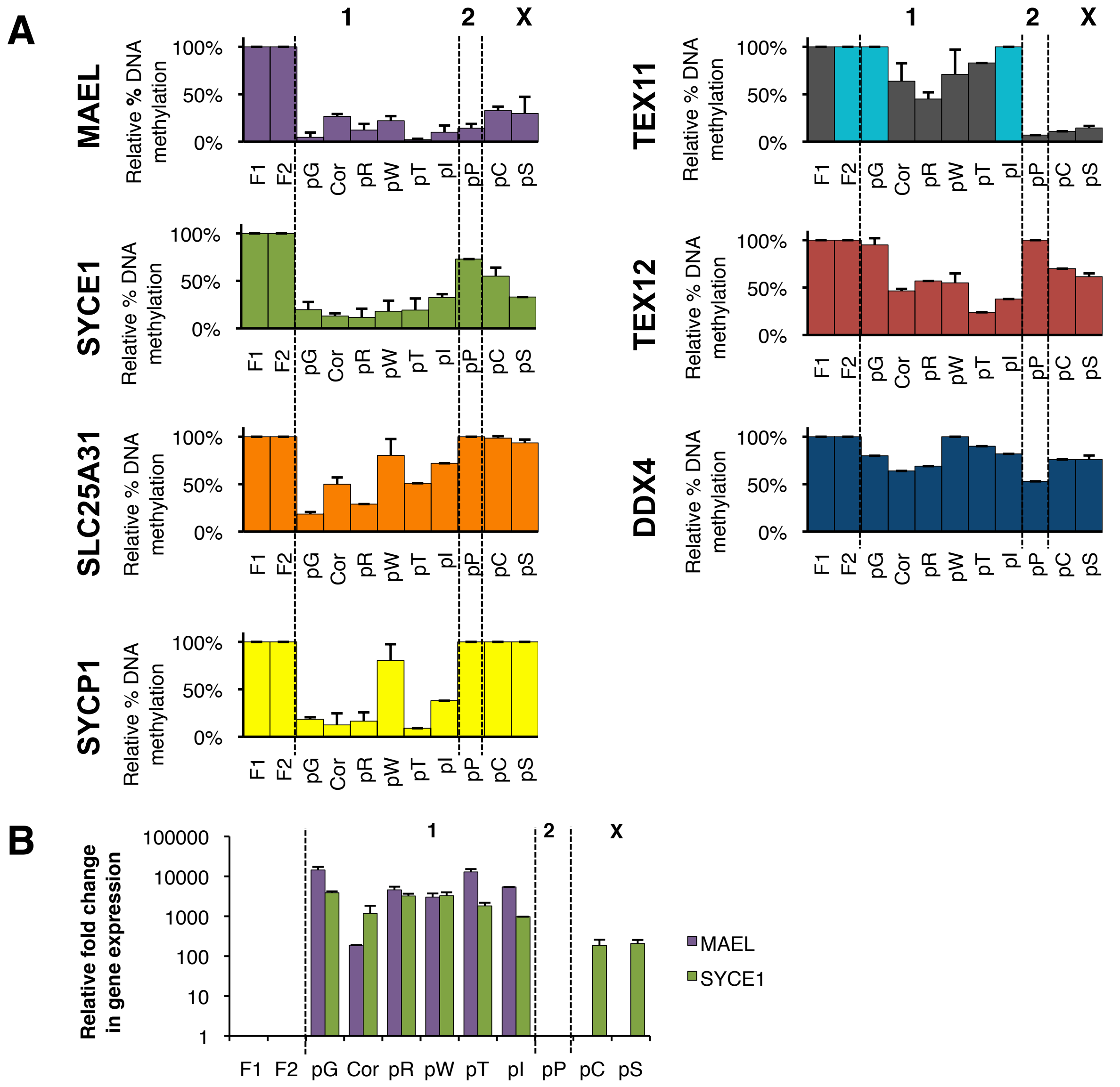

Additional File 7C. Relative promoter methylation and expression analysis of germline genes in patient-derived fibroblasts. (A) DNA methylation was assessed as in Figure 2. For the X-linked gene TEX11, female patients are indicated as black bars. (B) Expression levels of MAEL (purple bars) and SYCE1 (green bars) were assessed by qRT-PCR, normalized to U6 snRNA levels and presented as fold change relative to that of control fibroblasts from healthy donors (F1 and F2). Error bars represent standard error.
