# Supplementary material for: Germline genes hypomethylation and expression define a molecular signature in peripheral blood of ICF patients: implications for diagnosis and etiology
Source: Orphanet J Rare Dis. 2014 Apr 17;9:56. doi: 10.1186/1750-1172-9-56 (PMC4022050; doi:10.1186/1750-1172-9-56)
Supplement: Additional file 8 — Raw PCR data used to built DNA methylation histograms shown in Figure 2, and statistical analysis to compare healthy controls and ICF patients. [file 1750-1172-9-56-S8.pdf]

DNA Methylation levels in control samples: ΔCTm (CTm $_{AciI\ digest}$)							
Sample	MAEL	SYCE1	SLC25A31	SYCP1	TEX11	TEX12	DDX4
WB1	0.25 (23.43)	2.38 (26.78)	0.11 (23.90)	2.17 (25.75)	0.14 (25.40)	0.11 (26.54)	0.16 (23.66)
WB2	0.14 (23.53)	2.32 (26.61)	0.06 (23.42)	2.30 (26.39)	0.16 (24.21)	0.16 (26.51)	0.12 (23.63)
WB3	0.23 (23.45)	2.35 (26.60)	0.09 (23.79)	2.16 (26.39)	0.25 (24.15)	0.22 (26.96)	0.08 (24.75)
WB4	0.23 (23.99)	2.24 (27.28)	0.14 (23.93)	2.10 (25.57)	0.30 (24.10)	0.10 (26.23)	0.18 (23.60)
WB5	0.28 (23.08)	2.48 (26.89)	0.11 (23.95)	2.35 (26.31)	0.24 (23.83)	0.18 (27.05)	0.11 (23.74)
WB6	0.15 (23.54)	2.16 (26.54)	0.12 (24.09)	2.13 (26.56)	0.22 (24.34)	0.12 (26.55)	0.13 (23.76)
**DNA Methylation levels in ICF1 :ΔCTm (CTm AciI digest)**							
pG	2.40 (26.20)	4.84 (29.60)	1.38 (25.22)	4.88 (28.79)	0.05 (24.19)	0.06 (26.61)	0.05 (23.70)
pR	1.01 (26.31)	3.5 (28.23)	1.26 (25.97)	6.01 (31.63)	0.98 (25.55)	0.13 (28.01)	0.09 (23.83)
pW	0.92 (24.19)	5.81 (30.00)	2.07 (25.72)	4.46 (27.17)	1.25 (25.13)	0.54 (27.05)	0.07 (24.11)
pH	0.52 (23.50)	3.92 (27.98)	0.50 (23.89)	3.79 (27.00)	0.18 (23.79)	0.12 (26.41)	0.15 (23.70)
P1	0.62 (25.30)	2.85 (28.16)	0.64 (24.79)	2.97 (27.77)	0.91 (24.96)	0.13 (27.97)	0.06 (24.21)
pY	2.02 (26.87)	3.71 (29.05)	0.25 (24.60)	2.54 (26.09)	0.11 (24.90)	0.13 (27.00)	0.05 (24.34)
P2	3.00 (27.38)	3.86 (29.00)	2.37 (26.76)	5.71 (29.95)	0.17 (24.88)	0.04 (27.14)	0.03 (24.09)
P3	1.26 (25.31)	5.25 (30.10)	1.23 (25.65)	5.74 (29.92)	1.04 (24.60)	0.14 (27.14)	0.08 (24.02)
**P4**	**1.09 (25.18)**	**5.69 (30.41)**	**0.9 (25.30)**	**4.65 (29.11)**	**1.27 (25.07)**	**0.15 (27.63)**	**0.18 (24.38)**
P5	1.06 (25.11)	3.69 (29.13)	0.48 (24.80)	4.28 (28.55)	0.37 (25.22)	0.11 (27.58)	0.10 (24.51)
**DNA Methylation levels in ICF2 : ΔCTm (CTm $_{AciI\ digest}$)**							
P6	0.27 (23.80)	2.11 (26.93)	0.19 (23.84)	2.79 (26.87)	0.57 (25.04)	0.14 (26.74)	0.16 (24.00)
pD	0.23 (24.09)	2.07 (28.27)	0.18 (24.40)	2.97 (27.40)	0.54 (25.26)	0.35 (28.07)	0.11 (23.92)
pV	0.26 (24.17)	2.03 (26.72)	0.30 (24.26)	3.39 (27.52)	0.65 (25.11)	0.04 (26.65)	0.09 (23.91)
P7	0.17 (24.30)	1.97 (26.74)	0.12 (24.62)	2.43 (26.76)	0.13 (24.91)	0.09 (27.71)	0.10 (24.20)
P8	0.15 (24.14)	2.52 (25.73)	0.24 (24.37)	2.75 (26.57)	1.20 (25.35)	0.04 (26.97)	0.11 (24.47)
**DNA Methylation levels in ICFX : ΔCTm (CTm $_{AciI\ digest}$)**							
pC	0.11 (23.97)	2.27 (27.00)	0.07 (24.44)	2.63 (26.79)	1.25 (24.96)	0.10 (27.22)	0.06 (24.19)
pS	0.13 (23.96)	2.40 (26.40)	0.10 (24.00)	2.05 (25.63)	1.23 (24.48)	0.08 (27.52)	0.27 (24.17)
pN	4.72 (28.86)	4.51 (29.10)	1.24 (25.19)	6.26 (29.93)	1.40 (24.58)	0.31 (27.31)	0.25 (23.53)

Additional File 8A. Analysis of methylation at germline gene promoters in whole blood. Genomic DNA was extracted from whole blood of healthy donors (CTL), ICF1, ICF2 and ICFX patients. Methylation analysis levels were assessed by Methylation-Sensitive Restriction Assay, followed by qRT-PCR amplification of the AciI digested products using primers flanking at least two AciI sites within the promoter CpG island. A non-cutter NcoI control digest served to normalize data that are presented in Figure 2. For each sample, the differences in cycle threshold (ΔCT) between AciI and NcoI digests are indicated as an average value of 3 independent experiments.

$\Delta CTm = CTm_{AciI\ digest} - CTm_{NcoI\ digest}$). The raw cycle threshold (CT) values are indicated into brackets. The ΔCTm values obtained for diagnosis from of the young child diagnosed in Figure 3 are indicated in red.

DNA Methylation in control cell lines: ΔCT (CT_{AciI})							
Cell line	**MAEL**	**SYCE1**	**SLC25A31**	**SYCP1**	**TEX11**	**TEX12**	**DDX4**
IMR90	0.1 (24.93)	3.03 (27.95)	0.23 (24.95)	0.59 (24.46)	0.37 (25.79)	0.13 (28.82)	0.25 (25.77)
MCF-7	0.15 (24.64)	3.04 (29.92)	0.26 (25.46)	0.53 (23.78)	0.55 (25.63)	0.16 (29.51)	0.23 (25.68)
MCF-7 AZA	1.73 (27.24)	3.63 (31.23)	1.26 (27.13)	1.78 (2.25)	2.46 (28.27)	1.41 (32.05)	1.51 (27.58)

Additional File 8B. Raw PCR data used to build DNA methylation histograms shown in Additional File 5.
Genomic DNA was extracted from IMR90 fibroblasts, breast cancer cell line MCF-7 treated or not with the demethylating agent 5-azacytidine. For each sample, the differences in cycle threshold (ΔCT) between AciI and NcoI digests are indicated as indicated above (panel 8A). The raw cycle threshold (CT_{AciI}) values obtained after AciI digests are indicated into brackets.

Statistical data for DNA methylation analysis

MAEL				SLC25A31				TEX12			
Median ΔCTm				Median ΔCTm				Median ΔCTm			
CTL	ICF1	ICF2	ICFX	CTL	ICF1	ICF2	ICFX	CTL	ICF1	ICF2	ICFX
0.23	1.08	0.23	0.13	0.11	1.07	0.19	0.1	0.14	0.13	0.09	0.1
Standard error				Standard error				Standard error			
**0.06**	**0.81**	**0.05**	**2.66**	**0.03**	**0.7**	**0.07**	**0.67**	**0.05**	**0.14**	**0.13**	**0.13**
p value				p value				p value			
CTL vs ICF1	ICF1 vs ICF2	ICF2 vs CTL	ICFX vs CTL	CTL vs ICF1	ICF1 vs ICF2	ICF2 vs CTL	ICFX vs CTL	CTL vs ICF1	ICF1 vs ICF2	ICF2 vs CTL	ICFX vs CTL
**3,58E-03**	**7,41E-03**	**0.94**	**0.19**	**3,78E-03**	**0.01**	**8,28E-03**	**0.19**	**0.91**	**0.76**	**0.78**	**0.8**

SYCE1				SYCP1				DDX4			
Median ΔCTm				Median ΔCTm				Median ΔCTm			
CTL	ICF1	ICF2	ICFX	CTL	ICF1	ICF2	ICFX	CTL	ICF1	ICF2	ICFX
2.34	3.89	2.07	2.4	2.17	4.56	2.79	2.63	0.13	0.08	0.11	0.25
Standard error				Standard error				Standard error			
0.11	1.01	0.22	1.26	0.1	1.16	0.35	2.28	0.04	0.05	0.03	0.12
p value				p value				p value			
CTL vs ICF1	ICF1 vs ICF2	ICF2 vs CTL	ICFX vs CTL	CTL vs ICF1	ICF1 vs ICF2	ICF2 vs CTL	ICFX vs CTL	CTL vs ICF1	ICF1 vs ICF2	ICF2 vs CTL	ICFX vs CTL
**3,18E-04**	**4,43E-04**	**0.11**	**0.17**	**3,01E-04**	**9,74E-03**	**1,58E-03**	**0.14**	**0.07**	**0.24**	**0.43**	**0.24**

Additional File 8C. Statistical analysis of germline genes methylation profiles in whole blood of patients. The median values of ΔCTm shown in Additional File 8A and standard errors are indicated for each germline gene tested. Hypomethylation of *MAEL*, *SYCE1*, *SLC25A31*, *SYCP1* in ICF1 compared to CTL donors or ICF2 subjects is strongly significant ($p < 0.01$) and is highlighted in blue.

Statistical data for DNA methylation at TEX11						
Median ΔCTm						
CTL M	ICF1 M	ICF2 M	CTL F	ICF1 F	ICF2 F	ICFX F
0.2	0.17	0.55	0.24	1,04	1.20	1,25
Standard Errors						
0.07	0.12	0.23	0.02	0,16	NA	0,09
p value						
ICF1 M vs CTL M	ICF1 F vs CTL F	ICF2 M vs CTL M			ICFX F vs CTL F	
**0.64**	**2,18E-04**	**0.08**			**6,32E-04**	

Statistical data for DNA methylation at TEX11 within ICF2				
Median ΔCTm				
P6 (M)	pD (M)	pV (M)	P7 (M)	P8 (F)
0.63	0.65	0.57	0.13	1.14
Standard Errors				
0.31	0.09	0.07	0.17	0.42
p value female vs male				
P8 vs P6	P8 vs pD		P8 vs PV	P8 vs P7
**0.08**	**0.08**		**0.04**	**0.03**

Additional File 8D. Statistical analysis of TEX11 methylation in whole blood.

Left panel) The median and standard errors have been calculated in male (M) and female (F) healthy subjects (CTL), ICF1, ICF2 and ICFX patients. Hypomethylation of TEX11 in female versus male in healthy subjects and ICF patients was compared. Hypomethylation of TEX11 in female ICF1 and ICFX is strongly significant ($p < 0.01$) and is highlighted in blue. Right panel) Statistical significance of TEX11 hypomethylation among ICF2 patients was analyzed separately (only one female patient). Hypomethylation in the female ICF2 patient is significantly higher than in male pV and P7, and to a lesser extent P6 and pD, male patients ($p < 0.05$) and is highlighted in blue. NA; not applicable (only one patient in this category).
