# Supplementary material for: Germline genes hypomethylation and expression define a molecular signature in peripheral blood of ICF patients: implications for diagnosis and etiology
Source: Orphanet J Rare Dis. 2014 Apr 17;9:56. doi: 10.1186/1750-1172-9-56 (PMC4022050; doi:10.1186/1750-1172-9-56)
Supplement: Additional file 9 — Control experiment showing that expression of Maelstrom decreases with the number of passages in culture. [file 1750-1172-9-56-S9.pdf]

Velasco et al., Additional File 9

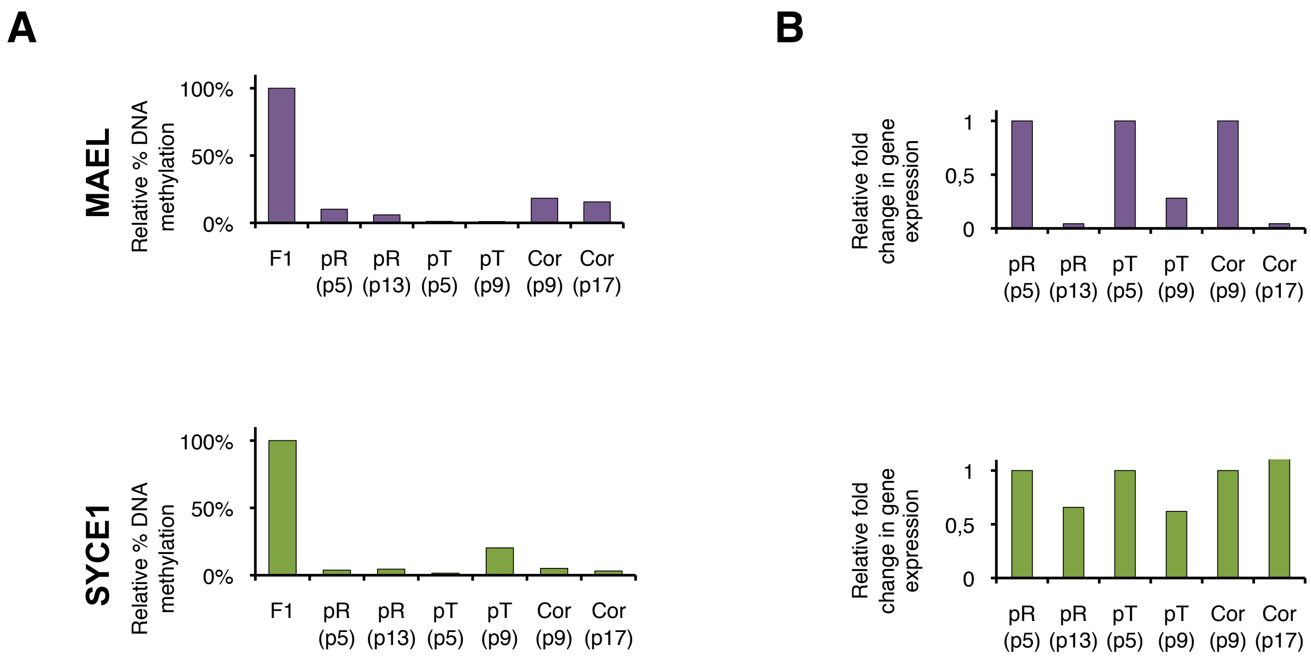

Additional File 9. Relative DNA methylation and expression analysis of MAEL and SYCE1 in primary fibroblasts derived from ICF patients at different passages in culture. (A) DNA methylation and (B) expression analysis were performed as in legend of Figure 1 and Figure 2. The number of passages are indicated into brackets.
