# Supplementary material for: Germline genes hypomethylation and expression define a molecular signature in peripheral blood of ICF patients: implications for diagnosis and etiology
Source: Orphanet J Rare Dis. 2014 Apr 17;9:56. doi: 10.1186/1750-1172-9-56 (PMC4022050; doi:10.1186/1750-1172-9-56)
Supplement: Additional file 10 — DNA methylation analysis at TEX12 and DDX4 promoters showing that their dependency on DNMT3B for methylation and silencing in murine cells is not conserved in humans. [file 1750-1172-9-56-S10.pdf]

Velasco et al., Additional File 10

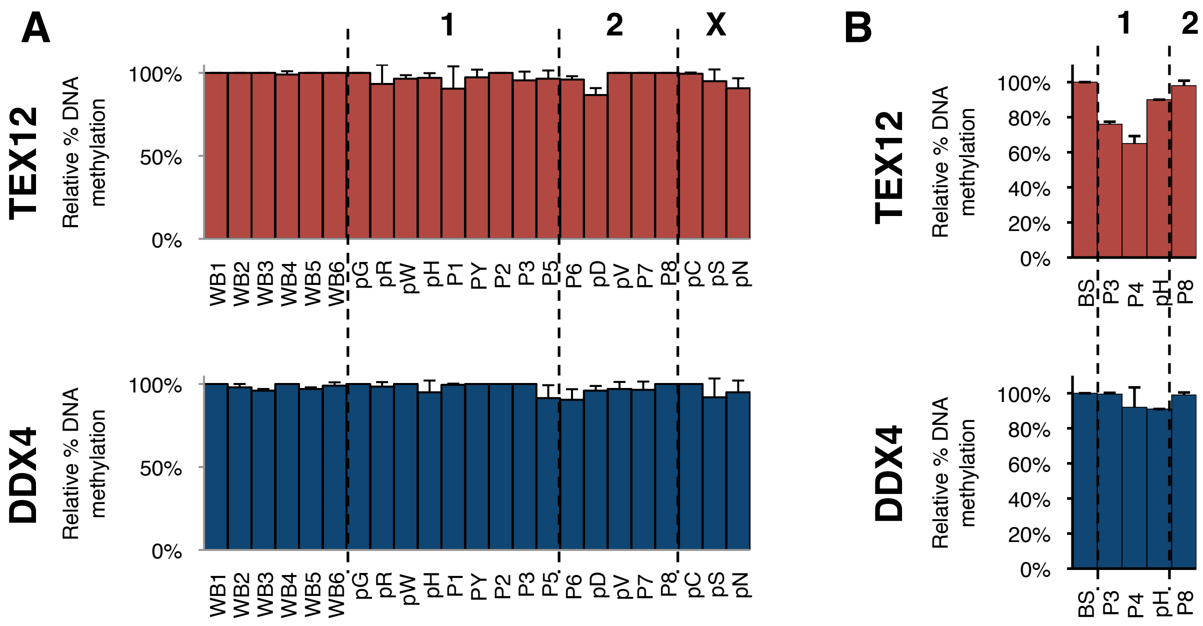

Additional File 10. Analysis of relative promoter methylation at TEX12 and DDX4 germline genes in whole blood and buccal swabs of ICF patients. Methylation analysis in whole blood (A) and buccal swabs (B) was assessed as in Figure 2. Error bars represent standard error. WB, Control whole blood from healthy donors; BS, control buccal swab.
